# Interaction between *Wolbachia pipientis* and *Leishmania infantum* in heartworm infected dogs

**DOI:** 10.1186/s13071-023-05662-y

**Published:** 2023-02-27

**Authors:** Maria Stefania Latrofa, Ilaria Varotto-Boccazzi, Viviane Noll Louzada-Flores, Roberta Iatta, Jairo Alfonso Mendoza-Roldan, Xavier Roura, Andrea Zatelli, Sara Epis, Claudio Bandi, Domenico Otranto

**Affiliations:** 1 Department of Veterinary Medicine, University of Bari, Bari, Italy; 2 Department of Biosciences, University of Milan, Milan, Italy; 3 Interdisciplinary Department of Medicine, University of Bari, Bari, Italy; 4 Hospital Clínic Veterinari, Universitat Autònoma de Barcelona, Barcelona, Spain; 5 Pediatric CRC ‘Fondazione Romeo ed Enrica Invernizzi’, University of Milan, Milan, Italy; 6 Faculty of Veterinary Sciences, Bu-Ali Sina University, Hamedan, Iran

**Keywords:** Canine, Dirofilariosis, TNFα, IFNγ, Immune response, IL-4, IL-6, IL-10, Leishmaniosis, *Wolbachia*

## Abstract

**Background:**

*Wolbachia* is a Gram-negative endosymbiont associated with several species of arthropods and filarioid nematodes, including *Dirofilaria immitis*. This endosymbiont may elicit a Th1 response, which is a component of the immunity against *Leishmania infantum.*

**Methods:**

To investigate the interactions between *Wolbachia* of *D. immitis* and *L. infantum* in naturally infected dogs and cytokine circulation, dogs without clinical signs (*n* = 187) were selected. Dogs were tested for microfilariae (mfs) by Knott, for female antigens of *D. immitis* by SNAP, and for anti-*L. infantum* antibodies by IFAT and assigned to four groups. Dogs of group 1 (G1) and 2 (G2) were positive for *D. immitis* and positive or negative to *L. infantum*, respectively. Dogs of group 3 (G3) and 4 (G4) were negative to *D. immitis* and positive or negative to *L. infantum*, respectively. *Wolbachia* and *L. infantum* DNA was quantified by real-time PCR (qPCR) in dog blood samples. A subset of dogs (*n* = 65) was examined to assess pro- and anti-inflammatory cytokine production using an ELISA test.

**Results:**

Of 93 dogs positive to *D. immitis* with circulating mfs, 85% were positive to *Wolbachia*, with the highest amount of DNA detected in G1 and the lowest in dogs with low mfs load in G1 and G2. Among dogs positive to *L. infantum*, 66% from G1 showed low antibody titer, while 48.9% from G3 had the highest antibody titer. Of 37 dogs positive to *Wolbachia* from G1, 26 (70.3%) had low antibody titers to *L. infantum* (1:160). Among cytokines, TNFα showed the highest mean concentration in G1 (246.5 pg/ml), IFNγ being the one most represented (64.3%). IL-10 (1809.5 pg/ml) and IL-6 (123.5 pg/ml) showed the highest mean concentration in dogs from G1. A lower percentage of dogs producing IL-4 was observed in all groups examined, with the highest mean concentration (2794 pg/ml) recorded in G2.

**Conclusion:**

Results show the association of *D. immitis* and *Wolbachia* with the lower antibody titers of *L. infantum* in co-infected dogs, suggesting the hypothesis that the endosymbiont may affect the development of the patent leishmaniosis. However, due to the limitations associated with the heterogeneity of naturally infected dogs in field conditions, results should be validated by investigation on experimental models.

**Graphical Abstract:**

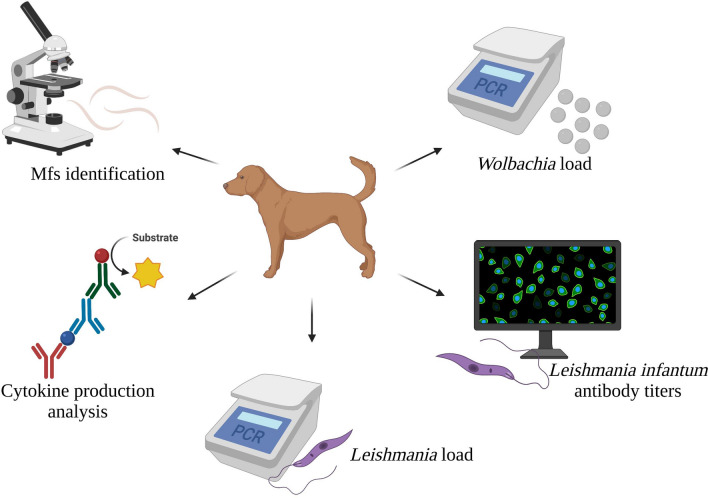

## Background

The endosymbiotic relationship described between *Wolbachia* and filarioid nematodes (families Onchocercinae, Dirofilariinae and Splendidofilariinae) has important implications in the biological processes of reproduction, development, molting and embryogenesis of filarioids [1–4]. *Dirofilaria immitis*, the causative agent of the canine heartworm disease (HWD), harbors *Wolbachia* endosymbiont in all its developmental stages, from adult to microfilariae (mfs) [5]. In addition, clinical studies indicate that *Wolbachia* is released into the bloodstream of hosts after death of *D. immitis* [6], also because of filaricidal treatment [7, 8]. Furthermore, *Wolbachia* associated to *D. immitis* infection may increase the severity of the clinical signs of HWD by triggering inflammatory response [7, 9–11]. As observed in other filarioids, such as *Onchocerca volvulus* [12], *D. immitis* may stimulate Th2 anti-inflammatory response, while *Wolbachia* triggers the Th1 pro-inflammatory response [10, 13, 14]. The latter is likely due to the effect that *Wolbachia* surface protein (WSP) and other molecules exert on antigen presenting cells and cytokine production [6, 7, 12, 15–18]. In addition, in vitro experiment using *Asaia*-WSP engineered bacterium was shown to elicit the Th1 cellular response against *Leishmania infantum*, with a protozoal killing effect [19].

Given the sympatric occurrence of *L. infantum* and *D. immitis* in many geographical areas around the world [20–24], the association of both vector-borne pathogens is of major interest from a diagnostic and clinical perspective [25]. For example, the prevalence of *D. immitis* infection increased in areas of southern Europe, where canine leishmaniosis (CanL) was historically endemic, as a consequence of pets traveling with their owners (i.e. from endemic to previously non-endemic regions) and changes in arthropod vector ecology and distribution [20, 21, 24, 26–30]. This overlapping geographical distribution of *D. immitis* and *L. infantum* led to potential immune interactions [20, 21, 24, 27, 28]. Indeed, the prevalence of dogs without clinical signs [31] depends on the balance between Th1 and Th2 immune response [32–35], which may be regulated by a plethora of factors (e.g. animal genetic, health and nutritional status, concurrent infections) [36]. In this context, the clinical manifestations of a patent leishmaniosis in dogs co-infected by *D. immitis* may be affected by the presence of *Wolbachia* endosymbiont [21, 27, 32]. Thus, the aim of this study was to investigate the interactions of *Wolbachia* in dogs naturally infected by *D. immitis* and/or *L. infantum* and to assess the relationship between pathogen infection and cytokine circulation.

## Methods

### Enrolled animals, parasitological and serological examination

A total of 187 dogs presenting no apparent clinical signs were selected to reduce other factors that could interfere with the analyses. All dogs, living in two municipal shelters in southern Italy (Lecce: 40.419326N, 18.165582E; Casarano 40.0126N, 18.1606E) were subjected to physical examination to establish their health status. Among them, a cohort of dogs (*n* = 84) was enrolled from a previous study aiming to control *D. immitis* and *L. infantum* infection [24].

Blood samples were collected from either the cephalic or jugular veins and placed in a K3 EDTA tube (2 ml) and in a clot activator tube (5 ml) to obtain serum after centrifugation (15 min at 1500 × *g*). Individual blood samples were screened by modified Knott test for the detection of mfs of *D. immitis*, as previously described [37]. The mfs identification was performed measuring the body length and width of specimens and by morphological analysis of the front end and the tail according to [38], using a digitally captured image software LAS V4.5 (Leica Microsystems). The count of the mfs (2 × 50 μl) was based on the average of the counting in the two slides. Dogs with a mfs load ≥ 600 mfs/20 μl were considered highly microfilaremic [24]. Serum samples were tested for the antigen detection of adult females of *D. immitis* by SNAP® 4Dx® Plus Test (IDEXX Laboratories, Inc.), according to the manufacturer's instructions.

To assess the exposure of dogs to *L. infantum* infection, sera were tested by immunofluorescence antibody test (IFAT) using as antigen the promastigotes of *L. infantum* zymodeme MON-1, as previously described [39]. Samples were considered positive when they produced a clear cytoplasmic and membrane fluorescence of promastigotes from a cut-off dilution of 1:80. Positive sera were titrated by serial dilutions until negative results were obtained.

Subsequently, all dogs were divided into four groups based on their positivity/negativity for mfs of *D. immitis* and to anti-*Leishmania* antibodies. Specifically, dogs positive for mfs and positive and/or negative to IFAT were grouped in G1 (*n* = 47) and G2 (*n* = 46), respectively, while dogs negative for mfs and for female antigens of *D. immitis* and positive or negative to IFAT were included in G3 (*n* = 47) and G4 (*n* = 47), respectively (Table 1).

**Table 1:** Dogs included in the study divided according to their positivity/negativity to *Dirofilaria immitis* and *Leishmania infantum*, microfilariae (mfs) load, IFAT titer and  positivity for *Wolbachia *and* L. infantum* by qPCR

Groups		Dogs	Knott test mfs load	Snap test	qPCR *Wolbachia*		IFAT *L. infantum* *n* dogs; titer	qPCR *L. infantum*	
					*n* dogs Pos (%; 95% CI)	*Ct*		*n* dogs Pos (%; 95% CI)	*Ct*
						min–mean–max			min–mean–max
G1	Dim + /Leis +	18	5 < low ≤ 200	Na	11 (61.1; 0.357–0.827)	29.2–33.1–37.1	*n* = 7; 1:80	1 (5.5; 0.001–0.273)	37.8
							*n* = 2; 1:160		
							*n* = 4; 1:320		
							*n* = 3; 1:640		
							*n* = 2; 1:2560		
		10	200 < medium ≤ 300		7 (70; 0.348–0.933)	26.6–31.7–35.3	*n* = 7; 1:80	1 (10; 0.003–0.445)	33.9
							*n* = 2; 1:320		
							*n* = 1; 1:640		
		19	High ≥ 600		19 (100; 0.824–1.000)	22.1–29.5–35.4	*n* = 11; 1:80	4 (21; 0.061–0.456)	24.7–30.7–36.5
							*n* = 4; 1:160		
							*n* = 2; 1:320		
							*n* = 2; 1:1280		
Total		47			37 (78.7; 0.643–0.893)			6 (12.8; 0.048–0.257)	–
G2	Dim + /Leis−	20	1 < low ≤ 200	Na	16 (75; 0.563–0.943)	28.8–32.9–37.4	Neg	Neg	Na
		11	200 < medium ≤ 300		11 (100; 0.715–1.000)	28.3–30.6–32.8			
		15	high ≥ 400		15 (100; 0.782–1.000)	28.4–31.1–35.6			
Total		46	–		42 (91.3; 0.792–0.976)		–	–	–
G3	Dim −/Leis +		Neg	Neg	Na	Na	*n* = 15; 1:80	2 (4.2; 0.005–0.145)	32.59–32.8–33.1
							*n* = 9; 1:160		
							*n* = 6; 1:320		
							*n* = 8; 1:640		
							*n* = 6; 1:1280		
							*n* = 3; 1:2560		
Total		47	–	–	–	–	–	2 (4.2; 0.005–0.145)	–
G4	Dim −/Leis −	47	Neg	Neg	Na	Na	Neg	Neg	Na
Total		187	–		79 (85; 0.760–0.915)			8 (4.3; 0.019–0.083)	

Minimum, mean and maximum of threshold value are indicated. *C*_*t*_ = threshold value, *95% CI* = 95% confidence level

In addition, a group of dogs (*n* = 65), selected based on their negativity and/or positivity to IFAT with a titer ≥ 1:160 and for mfs, were examined by analyzing the production of pro-inflammatory cytokines such as tumor necrosis factor α (TNFα) and interferon-gamma (IFNγ) and of anti-inflammatory cytokines such as interleukin-4 (IL-4), interleukin-6 (IL-6) and interleukin-10 (IL-10) using an ELISA kit, (Biolegend, USA, and Thermo Fisher, USA) (Table 2). The positivity of the sera was determined based on specific standard curves.

**Table 2:** Prevalence of cytokine production, calculated as the percentage of dogs from each group producing one cytokine, divided according to groups

Groups	qPCR *Wolbachia*	Cytochine production									
	n dogs Pos (%)	TNFα		IFNγ		IL-10		IL-4		IL-6	
		Concentration (pg/ml)	Prevalence	Concentration (pg/ml)	Prevalence	Concentration (pg/ml)	Prevalence	Concentration (pg/ml)	Prevalence	Concentration (pg/ml)	Prevalence
		min–mean–max	*n* dogs Pos (%; 95%CI)	min–mean–max	*n* dogs Pos (%; 95%CI)		*n* dogs Pos (%; 95%CI)	min–mean–max	n dogs Pos (%; 95%CI)	min–mean–max	n dogs Pos (%;95%CI)
G1 (n = 14)	12 (85.7)^6,7,8^	44.8–246.5–649.4	4 (28.6; 0.084–0.581)^1,8^	3.6–88.2–475	9 (64.3; 0.351–0.872)^a,2^	5.97–1809.5–4120	10 (71.4; 0.419–0.916)^1,3^	77.2	1 (7.1; 0.002–0.339)^2,3,6^	3.01–123.5 -392.6	5 (35.7; 0.128–0.649)^7^
G2 (n = 14)	14 (100)	2.27 – 65.1 -172.9	6 (42.8; 0.177–0.711)	13.3–105.4–265.1	7 (50; 0.230–0.770)^b^	19.1–507.8–1440	7 (50; 0.230–0.770)	501.1–2794.5–5739.5	4 (28.5; 0.084–0.581)	5.05–30.2–105	7 (50; 0.230–0.770)
G3 (n = 14)	N/A	–	–	0.65–97.23–617.6	7 (50; 0.230–0.770)^b^	50.4–677.4–2644.8	6 (42.8; 0.177–0.711)	62.5–254.9–480.4	4 (28.5; 0.084–0.581)	6.1–29.7–67.2	7 (5 0:0.230–0.770)
G4 (n = 14)	N/A	8.1- 13.3–18.6	2 (14.3; 0.018–0.428)4	3.6–3.7–23.7	2 (14.2; 0.018–0.428)^a,b,4^	81.9–863.8–3320	8 (57.1; 0.289–0.823)^4,5^	62.5–111.7–160.9	2 (14.3; 0.018–0.428)^5^	5.05–13.9–22.3	5 (35.7; 0.128–0.649)
Total (n = 56)	26 (46.4)	–	12 (21.4; 0.116–0.344)	–	25 (44.6; 0.313–0.585)	–	31 (55.4; 0.415–0.687)	–	11 (19.6; 0.102–0.324)	–	24 (42.9; 0.297–0.568)

Minimum, mean and maximum of concentration (pg/ml) are indicated. Statistically significance differences in prevalence are marked with equal superscript letters. *P*-value < 0.05. *95% CI* = 95% confidence level.

### Genomic DNA blood extraction and molecular procedures

Genomic DNA (gDNA) was extracted from each blood sample using the GenUPgDNA commercial kit (Biotechrabbit GmbH, Hennigsdorf, Germany) according to the manufacturer’s instructions. All samples were tested for *L. infantum* kDNA minicircle by real time-PCR (qPCR) using the primers, probes and cycle protocol described elsewhere [40]. gDNA from *L. infantum* isolate cultured zymodeme MON-1 was used as positive control, whereas gDNA extracted from blood sample from a healthy dog was used as negative controls.

Samples were tested for *Wolbachia* of *D. immitis* by qPCR using primers (111 bp, W*Diro*.ftsZ.490-F/W*Diro*.ftsZ.600-R) and probe w*Dimm*.ftsZ.523p (6FAM-CGTATTGCAGAGCTCGGATTA-TAMRA) targeting the *ftsZ* gene as previously described [41] with minor modifications.

Briefly, all qPCR reactions were carried out in a final volume of 20 μl, consisting of 10 μl IQ Supermix (Bio-Rad Laboratories, Hercules, CA, USA), 6 μl diethyl pyrocarbonate (DEPC)-treated pyrogen-free DNase/RNase-free water (Invitrogen, Carlsbad, CA, USA), 2.5 μl of template DNA (except no-template control), primers and probe at 50 μM and 20 μM concentration, respectively. The run protocol consisted of a hot-start at 95 °C for 3 min and 40 cycles of denaturation (95 °C for 5 s) and annealing-extension (60 °C for 30 s). The qPCR was performed in a CFX96 Real-Time System (Bio-Rad Laboratories, Inc., Hercules, CA, USA), and the increase in the fluorescent signal was registered during the extension step of the reaction and analyzed using CFX Manager Software, version 3.1 (Bio-Rad Laboratories, Inc., Hercules, CA, USA). All the samples were tested in duplicate, and DNA of adult of *D. immitis* and that from blood samples of pathogen-free dogs were used as positive and negative controls. The positivity for *Wolbachia* was established based on the threshold cycle (*C*_*t*_) value up to 38.5.

### Statistical analysis

Associations between infections and variables were assessed through univariate analysis. Exact binomial test established the confidence intervals (CI) with 95% confidence level. The Chi-square *χ*^2^ test was used to compare percentages of positivity among categories of the same independent variables. Collinearity among independent variables was assessed using Pearsonʼs correlation coefficient. A *P*-value < 0.05 was considered as statistically significant. Statistical analyses were performed using StatLib for Windows (version 13.0, SPSS, Inc., Chicago, IL, USA) Quantitative Parasitology 3.0 [42] and GraphPad Prism 8 software.

### Results

Of 93 mfs-positive dogs, 79 (85%) tested positive for *Wolbachia* DNA (Table 1), with 37 from G1 (46.8%; 95% CI: 0.355–0.584) and 42 from G2 (53.2%; 95% CI: 0.416–0.645). The highest amount of *Wolbachia* DNA (*C*_*t*_ value 22.1) was detected in a dog from G1 (mfs load ≥ 600) and the lowest (*C*_*t*_ value 37.4) in dogs with low mfs load (i.e. 1 ≤ mfs ≤ 200) in both G1 and G2 (Table 1). No statistically significant difference in *Wolbachia* prevalence was observed between dogs from G1 and G2, though an overall higher value (37.6%) was recorded in dogs with a *Wolbachia* *C*_*t*_ values ranging from 25 to 30 (Table 3). In G1, a statistical difference was observed between dogs with *Wolbachia* *C*_*t*_ value of 25–30 vs. 30–34 (*χ*^2^ = 6, *P* < 0.0138) and vs. *C*_*t*_ > 35 (*χ*^2^ = 12.3, *P* = 0.0004) having a high mfs load and between low vs. high mfs score (*χ*^2^ = 10.64, *P* = 0.011) (Table 3). In G2, a statistically significant difference (*χ*^2^ = 9.8, *P* = 0.00178) was observed between dogs with *Wolbachia* *C*_*t*_ value of 25–30 and 30–34 vs. > 35, having high mfs load (Table 3).

**Table 3:** Prevalence of *Wolbachia* in dogs positive for *Dirofilaria immitis* divided according to the microfilaria load and the range of *C*_*t*_ value of qPCR

Groups		Knott test	*n* dogs	qPCR *Wolbachia*							
		mfs load		*C*_*t*_ = 0	Prevalence (%); 95% CI	25 ≤ *C*_*t*_ ≤ 30	Prevalence (%); 95% CI	30 < *C*_*t*_ ≤ 34	Prevalence (%); 95% CI	*C*_*t*_ > 35	Prevalence (%); 95% CI
G1	Dim + /Leis +	5 ≤ low ≤ 200	18	7	38.9; 0.173–0.643	2^d^	11.1; 0.014–0.347	4	22.2; 0.064–0.476	5	27; 0.097–0.535
		200 < medium ≤ 300	10	3	30; 0.067–0.652	3	30; 0.067–0.652	1	10; 0.003–0.445	3	30; 0.067–0.652
		High ≥ 600	19	0	–	12^c,d^	63.2; 0.384–0.837	5^c^	26.3; 0.091–0.51	2^c^	30; 0.067–0.652
	Tot		47	10	21.3; 0.107–0.357	17	36.2; 0.227–0.515	10	21.3; 0.107–0.357	10	21.3; 0.107–0.357
G2	Dim + /Leis−	1 ≤ low ≤ 200	20	5	25%; 0.087–0.491	5	25; 0.087–0.491	6	30; 0.119–0.543	4	20; 0.057–0.437
		200 < medium ≤ 300	11	0	–	5	54.5; 0.234–0.833	6	54.5; 0.234–0.833	0	0
		High ≥ 400	15	0	–	8^e^	53.3; 0.266–0.787	6^e^	40; 0.163–0.677	1^e^	6.7; 0.002–0.319
	Tot		46	5	10.9; 0.036–0.236	18	39.1; 0.251–0.546	18	39.1; 0.251–0.546	5	10.9; 0.036–0.236
Total			93	15	16.1; 0.093–0.252	35^a^	37.6; 0.278–0.483	28^b^	30.1; 0.210–0.405	15^a,b^	16.1; 0.093–0.252

*C*_*t*_ = threshold value, *95% CI* = 95% confidence level. Statistically significant differences in prevalence are marked with equal superscript letters. *P*-value < 0.05

Of 94 dogs positive for anti-*Leishmania* antibodies, 31 were from G1 (66%; 95% CI: 0.507–0.791) with low antibody titers (up to 1:160), followed by 16 dogs (34%; 95% CI: 0.209–0.49) with higher titers (from 1:320 to 1:2560). In G3, 23 dogs (48.9%; 95% CI: 0.341–0.639) had a high antibody titer (from 1:320 to 1:2560) (Table 1). The qPCR screened eight positive dogs of 187 blood samples examined for kDNA of *L. infantum* (4.3%) with a minimum and maximum *C*_*t*_ value (min = 24.7, max = 37.8) detected for dogs from G1 (Table 1).

Of 37 dogs positive for *Wolbachia* in G1*,* 26 (70.3%; 95% CI: 0.530–0.841) had low antibody titers (*n* = 20 1:80, *n* = 6 1:160) against *L. infantum* (Table 4). A statistical difference was observed between dogs showing the highest amount of *Wolbachia* DNA (*C*_*t*_ value range of 25–30) and those with antibody titers ranging from 1:320 to 1:2560 (*χ*^2^ = 6.8, *P* = 0.0089) (Table 4).

**Table 4:** *Wolbachia*-positive dogs divided according to *C*_*t*_ value of endosymbiont DNA and *Leishmania infantum* positivity to different tests

Group	*n* dogs Pos (%)	qPCR *Wolbachia*	IFAT *L. infantum*						qPCR *L. infantum*
			*n* dogs Pos (%)						
			1:80	1:160	1:320	1:640	1:1280	1:2560	*n* dogs Pos (%)
G1	15 (40.5)^a,c^	25 ≤ *C*_*t*_ < 30	10 (66.7)	(1 (6.7)	2 (13.3)	0	2 (13.3)	0)^a^	3 (20)^c^
	13 (35.1)^b,d^	30 ≤ *C*_*t*_ < 34	5 (38.5)^b^	2 (15.4)	3 (23.1)	2 (15.4)	0	1 (7.7)	3 (23.1)^d^
	9 (24.3)	34 ≤ *C*_*t*_ < 35	5 (55.5)	3 (33.4)	0	1 (11.1)	0	0	0
Total	37		20 (54.1)	6 (16.2)	5 (13.5)	3 (8.1)	2 (5.4)	1 (2.7)	6 (16.2)

**Fig. 1:**
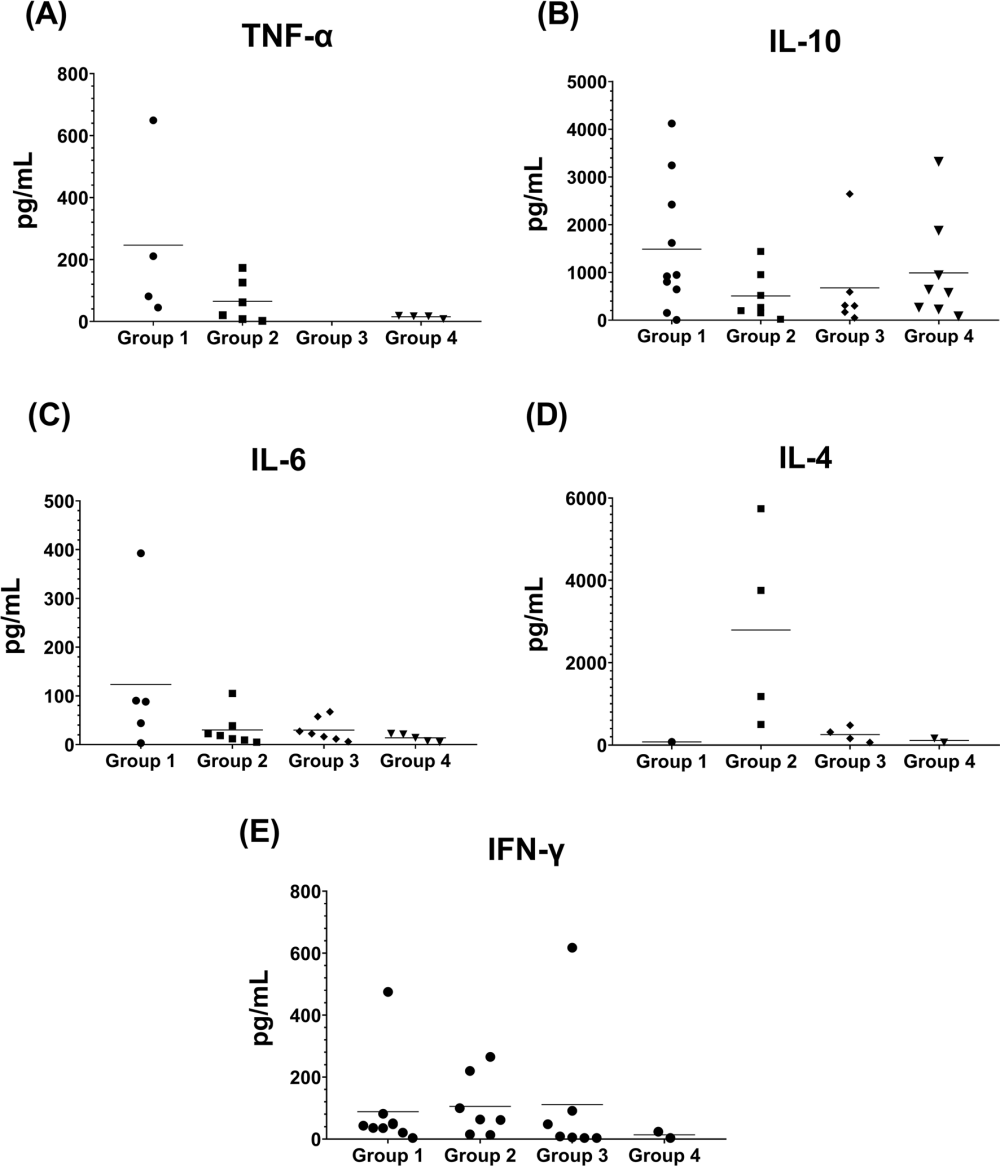
Concentrations of **A** tumor necrosis factor-α (TNFα), **B** interleukin-10 (IL-10), **C** interleukin-6 (IL-6), **D** interleukin-4 (IL-4) and **E** interferon-γ (IFNγ) in sera from dogs positive for *Leishmania infantum* and *Dirofilaria immitis* (Group 1), positive only for *Dirofilaria immitis* (Group 2), positive only for *Leishmania infantum* (Group 3) and negative to both pathogens (Group 4)

IL-10 (55.4%) and IFNγ (44.6%) were the most prevalent cytokines produced throughout dogs examined (Table 2 and Fig. 1). In particular, the percentage of dogs producing TNFα was higher in G2 (42.8%) than G1 (28.6%) and G4 (14.3%). However, the highest mean of TNFα concentration was detected in G1 (246.5 pg/ml) and the lowest in G2 (65.1 pg/ml) and G4 (13.3 pg/ml) (Table 2, Fig. 1). A statistically significant difference (χ^2^ = 7.3, *P* = 0.007) was recorded analyzing the percentages of IFNγ-positive sera between dogs from G1 (64.3%) and G4 (14.2%), with the same percentage of positive dogs in G2 and G3 (50%) (Table 2). The highest mean of concentration of IFNγ production was observed in G2 (105.4 pg/ml) and G3 (97.23 pg/ml) (Table 2, Fig. 1). The highest percentage of dogs producing IL-10 (71.4%) was observed in G1 (mean concentration of 1809.5 pg/ml) followed by G4 (57.1%) and G3 (42.8%) (Table 2). The lowest percentage of dogs producing IL-4 was observed in G1 (7.1%), G2 and G3 (28.5%) (Table 2, Fig. 1). The highest mean concentration of IL-4 was observed in G2 (2794.5 pg/ml). IL-6 showed the same percentage of positivity in G1 and G4 (35.7%) and in G2 and G3 (50%), respectively, with the highest mean concentration registered in G1 (123.5 pg/ml) and a similar concentration observed in G2 (30.2 pg/ml) and G3 (29.7 pg/ml) (Table 2, Fig. 1).

Except for IFNγ, no statistically significant differences were observed in cytokine production between groups, but within groups. In particular, in G1 a statistically significant difference was observed between dogs producing TNFα vs. IL-10 (*χ*^2^ = 5.1, *P* = 0.023), IFNγ vs. IL-4 (*χ*^2^ = 9.9, *P* = 0.001), IL-10 vs. IL-4 (*χ*^2^ = 12.1, *P* = 0.005) and IL-4 vs. IL-6 (*χ*^2^ = 17.4, *P* = 0.00003). Similarly, in G4 a statistically significant difference was observed between IFNγ and TNFα vs. IL-10 and between IL-10 vs. IL-4 (*χ*^2^ = 5.6, *P* = 0.018). No statistically significant difference was observed in cytokine production within groups G2 and G3 (Table 2). In addition, a statistically significant difference was observed in G1 between dogs positive for *Wolbachia* vs. TNFα (*χ*^2^ = 9.3, *P* = 0.022), IL-4 (*χ*^2^ = 17.4, *P* = 0.00003) and IL-6 (*χ*^2^ = 7.3, *P* = 0.0067) (Table 2).

## Discussion

Data herein presented suggest that *Wolbachia*, associated with *D. immitis*, may affect the immune response against *L. infantum* of naturally co-infected dogs through a stimulatory or immune-suppressive mechanism [4]. Indeed, the high molecular prevalence of *Wolbachia* in co-infected dogs (78.7%) is coherent with the hypothesis that this endosymbiont might control the *Leishmania* infection. Overall, the prevalence of *Wolbachia* observed in co-infected dogs is in line with data described in a previous study [21], where the endosymbiont was detected in 68.8% dogs from Portugal. Nevertheless, the high amount of *Wolbachia* DNA in relationship to the mfs load (i.e. mfs > 600; *C*_*t*_ 25–30) exclusively observed in co-infected dogs (63.2%) differed from a previous study from Spain, where *Wolbachia* was more frequently detected in microfilaraemic dogs not infected with *L. infantum* [27]. In addition, in this previous study, an increased severity of clinical leishmaniotic signs was observed in microfilaraemic dogs with a lower prevalence of *Wolbachia* [27]. Furthermore, the role of the endosymbiont in stimulating a Th1 immune response was also suggested by the low number (3/11) of co-infected dogs with clinical signs, as previously described [21].

The role of *Wolbachia* in controlling the development of CanL may be highlighted by the high prevalence of co-infected dogs (70.3%) showing a low anti-*Leishmania* antibody titer (up to 1:160), likely due to the low parasitic load detected by qPCR in the blood [43], while a high value of IFAT titers was observed in most of dogs (48.9%) infected only with *L. infantum*.

Furthermore, the absence of clinical signs in dogs may be also determined by the stimulation of a Th1 immune response triggered by the endosymbiont bacterium by production of high levels of pro-inflammatory cytokines [12, 19, 21, 27, 44]. For example, the high *Wolbachia* amount recorded in co-infected dogs may trigger an elevated TNFα production (mean value 246.5 pg/ml), which was not recorded in dogs infected with only *L. infantum*. The strong effect of *Wolbachia* in producing this pro-inflammatory cytokine has also been demonstrated in an in vitro experiment [19], regardless of the presence of *L. infantum*. However, the absence of TNFα observed in dogs positive for *L. infantum* agrees with previous studies where the cytokine was detected only in a few dogs with active leishmaniosis [45, 46].

The *Wolbachia* amount detected in co-infected dogs, and consequently its effect on controlling the *L. infantum* infection, may also be supported by the high prevalence of dogs (64.3%) producing IFNγ, a similar mean cytokine concentration value being observed between these dogs (88.2 pg/ml) and those positive for *L*. *infantum* (97.23 pg/ml) or *D. immitis* (105.4 pg/ml). Accordingly, IFN-γ was associated with the absence or low antibody titer against *L. infantum* [47]. Besides TNFα and IFNγ, the detection of IL-4, IL-6 and IL-10 in infected dogs indicates a mixed Th1/Th2 immune response. Indeed, in previous studies using peripheral blood mononuclear cells (PBMCs) of dogs experimentally infected with *L. infantum* or stimulated with *L. infantum* antigen, an increase in IFNγ, IL-10 and IL-4 mRNA expression levels were recorded [35, 48, 49]. In addition, the expression of cytokines related to both Th1/Th2 responses was also detected in dogs naturally infected with *D. immitis* with circulating mfs associated to the presence of *Wolbachia* [10, 50]. All the above considerations may justify the high mean concentration (1809.5 pg/ml) of IL-10 recorded in co-infected dogs as well as its even production in dogs infected with *D. immitis* (507.8 pg/ml) or *L. infantum* (677.4 pg/ml). However, though IL-10 is considered a predictive parameter of the evolution of CanL and active visceral leishmaniosis in humans [51–53], other studies described the expression of this cytokine in dogs without clinical signs [48, 54–56]. Furthermore, the detection of IL-10 in uninfected dogs (863.8 pg/ml) agrees with the results described elsewhere [54], where the mRNA accumulation of this cytokine in dogs with severe disease was comparable with that of uninfected control dogs. Similarly, the detection of IL-4 in all groups of dogs examined is not surprising, considering that the role of this cytokine in the pathogenesis of CanL is still debated [54]. Indeed, in the current study a very low mean concentration (77.2 pg/ml) of IL-4 was detected in co-infected dogs compared with those recorded in *L. infantum* infected (254.9 pg/ml) or uninfected dogs (111.7 pg/ml). However, other studies also described these contrasting results, IL-4 being detected readily or not in *L. infantum* infected or asymptomatic dogs [48, 54, 57, 58]. The *Wolbachia* amount recorded in the co-infected dogs may have affected the IL-6 production (123.5 pg/ml) in these dogs. Indeed, the role of this endosymbiont in stimulation of IL-6 production was also supported by an in vitro experiment [19]**.** However, though IL-6 is generally regarded as a Th2 cytokine with disease progression, other studies described its protective role in some forms of leishmaniosis [59], thus indicating an imperfect fitting of this cytokine into the Th1/Th2 paradigm [45, 48, 60].

Overall, interpreting the cytokine expression profile in CanL is still problematic, and the differences observed regarding the role of cytokines may be due to the different methods used for the analyses (i.e. dosage by ELISA or evaluation of mRNA expression) [48, 54, 56]. In addition, immune response mediated by cytokines may be influenced by several variables (i.e. age, tissues examined) [35, 53]. Thus, the comprehensive knowledge of cytokine response and their interaction is a very crucial step to understand disease progression, mainly in co-infected dogs.

## Conclusions

Though the present work has some limitations, such as the low number of dogs included and their heterogeneity, and the lack of a follow-up study, the results presented suggest the involvement of *Wolbachia* in clinical leishmaniosis, pointing at a possible role of this endosymbiont in the modulation of the Th1 immune response. However, future studies based on the simultaneous combination of different approaches of analysis (i.e. expression of the mRNA vs. quantification of cytokine production) is mandatory.

## Data Availability

All data generated or analyzed during this study are included in this published article.

## Abbreviations

CanL: Canine leishmaniosis
Ct: Threshold cycle
qPCR: Real-time PCR
EDTA: Ethylenediamine tetraacetic acid
IFAT: Indirect immunofluorescent antibody test
CI: Confidence intervals
ELISA: Enzyme-linked immunosorbent assay
TNFα: Tumor necrosis factor α
IFNγ: Interferon-gamma
IL-4: Interleukin-4
IL-6: Interleukin-6
IL-10: Interleukin-10
